# Trazodone effectiveness in depression: impacts of trazodone extended release vs SSRIs on the health status and quality of life of patients with major depressive disorder

**DOI:** 10.3389/fphar.2024.1525498

**Published:** 2025-01-23

**Authors:** Marcin Siwek, Adrian Andrzej Chrobak, Anna Julia Krupa, Aleksandra Gorostowicz, Andrzej Juryk, Dominika Dudek

**Affiliations:** ^1^ Department of Affective Disorders, Jagiellonian University Medical College, Krakow, Poland; ^2^ Department of Adult Psychiatry, Jagiellonian University Medical College, Krakow, Poland

**Keywords:** trazodone, selective serotonin reuptake inhibitors, major depressive disorder, quality of life, EQ-5D-5L

## Abstract

**Introduction:**

Early research on the pharmacotherapy for major depressive disorder (MDD) has largely focused on symptomatic improvements, whereas this focus has shifted to functioning and quality of life in recent years. Studies have confirmed that antidepressants generally improve the functional outcomes in MDD, but very few works have compared the efficacies of specific drugs. The present work aims to compare the impacts of trazodone once-a-day extended-release (XR) vs selective serotonin reuptake inhibitors (SSRIs) on the health status and quality of life in MDD.

**Methods:**

Data were gathered from 180 subjects through a naturalistic observation study of trazodone effectiveness in depression (TED) and analyzed. The TED study participants received trazodone XR of SSRIs in flexible doses for 12 weeks. The health status and health-related quality of life (HRQoL) were evaluated using the EQ-5D-5L tool at baseline as well as 2, 4, 8, and 12 weeks.

**Results:**

At baseline, the subjects treated with trazodone XR vs SSRIs presented similar health status profiles and HRQoL values with respect to the mobility, self-care, and anxiety/depression dimensions along with lower scores for the usual activities, pain/discomfort, overall HRQoL, and health status. Both trazodone XR and SSRIs improved the health status and HRQoL of the MDD patients at all subsequent timepoints. Compared to SSRIs, trazodone XR provided greater improvements in terms of the self-care, usual activities, pain/discomfort, and anxiety/depression measures and more often improved participant overall health status and HRQoL. More participants reported mixed changes in their health status and HRQoL in the SSRI group than the trazodone XR group.

**Discussion:**

Health status and HRQoL improved in both treatment arms, with preferable scores in trazodone XR vs. SSRIs group.

## 1 Introduction

In earlier research on antidepressant drugs, the expectations of clinicians regarding the treatment outcomes for major depressive disorder (MDD) were largely focused on symptomatic response and remission. Consequently, most of the clinical trials on MDD and their meta-analyses report only the symptomatic outcomes (Cipriani et al., 2018; Kowalczyk et al., 2024; Rodolico et al., 2024). However, as shown by Zimmerman et al. (2006), the most important outcomes considered by patients for determining remission are the presence of positive mental health features, return to an individual’s usual normal self, and return to the usual level of functioning. Although there is a clear link between general symptomatic and functional improvements (Miller et al., 1998; Dennehy et al., 2014), some symptoms are crucial to functional recovery and quality of life, namely anhedonia (Cao et al., 2019), emotional blunting (Fagiolini et al., 2021; Kikuchi et al., 2024), and cognitive functioning (Bortolato et al., 2016; Chokka et al., 2019). The dynamics of the treatment effects are also important as early functional improvement is a predictor of later therapeutic success, which can be defined as both symptomatic and functional responses and remission (Soares et al., 2020). Hence, the perspectives on the pharmacotherapeutic goals of MDD have evolved from focusing solely on symptom resolution to acknowledging the importance of functional recovery (McIntyre et al., 2015) and restoration of the quality of life (Zimmerman and Lin, 2023). However, as reported by Kowalczyk et al. (2024), only 28% of the placebo-controlled randomized clinical trials (RCTs) on antidepressants for MDD have assessed functional remission, and none of these trials have considered the functional outcomes as the primary goals. In a systematic review of the health state utility values (HSUVs) of MDD published in 2021, it was reported that only 16 MDD trials incorporated assessment of measures that allow quantification of the health-related quality of life (HRQoL) (Brockbank et al., 2021). Hence, analyses of the impacts of antidepressants are crucial not only for MDD symptoms but also for the HRQoL because the available data show that the subpopulation of patients achieving symptomatic outcomes is not identical to the subpopulation achieving functional outcomes (Kennedy, 2022).

Nowadays, evidence supporting the effectiveness of antidepressants in improving the functional outcomes in MDD seems robust (Sheehan et al., 2017; Cao et al., 2022; de Beste et al., 2024). As reported by Cao et al. (2022), the highest efficacy was observed for duloxetine, followed by paroxetine, levomilnacipran, venlafaxine, quetiapine, desvenlafaxine, agomelatine, escitalopram, amitriptyline, bupropion, sertraline, vortioxetine, and fluoxetine. However, trials comparing particular antidepressants directly are scant.

Trazodone is a multimodal antidepressant with well-documented efficacy in MDD management (Albert et al., 2023; Fagiolini et al., 2023; Crapanzano et al., 2024). However, there is little data on its impacts on the functioning and quality of life of MDD subjects, with only one study comparing it with other antidepressants in this regard (Tellone et al., 2024). In previous works, we reported the effectiveness of trazodone extended release (XR; also known as once-a-day) in improving not only the overall depressive symptomatology but also anhedonia, anxiety, insomnia, and general functioning in particular in patients initiating MDD pharmacotherapy *de novo* as being comparable (and in some aspects favorable) to selective serotonin reuptake inhibitors (SSRIs) (Dudek et al., 2023; Siwek et al., 2023b); we also noted that trazodone XR treatment is effective in reducing in depression, anhedonia, anxiety, and insomnia while improving functioning in subjects with unsatisfactory responses to SSRIs (compared to those who received trazodone XR as a first-line drug) (Siwek et al., 2023a). The aim of the present work was to evaluate the effectiveness of trazodone XR vs SSRIs on the health status and quality of life in MDD.

## 2 Methods

### 2.1 Study design

The trazodone effectiveness in depression (TED) study was conducted as a 12-week, non-randomized, open-label trial to compare the effectiveness of flexibly dosed trazodone XR vs SSRIs in patients diagnosed with MDD and recurrent depressive disorder. Clinician- and patient-rated tools were applied to measure the severity of the following factors: depression (Montgomery–Åsberg Depression Rating Scale as well as clinician- and patient-rated Quick Inventory of Depressive Symptomatology as the primary endpoints of the study), anhedonia (Snaith–Hamilton Pleasure Scale), anxiety (Hamilton Anxiety Rating Scale), insomnia (Athens Insomnia Scale), and therapeutic effectiveness (Clinical Global Impression Scale). Evaluations were performed at baseline and after 2, 4, 8, and 12 weeks of treatment. The methodology used was identical to that described in an earlier work (Dudek et al., 2023; Siwek et al., 2023b). The present study was conducted in accordance with the guidelines of the Declaration of Helsinki and approved by the Bioethics Committee of Jagiellonian University in Krakow, Poland (approval no. 1072.6120.113.2021). All participants provided written informed consent for participation in this study.

### 2.2 Quality of life assessment

This analysis explored the effectiveness of trazodone XR and SSRIs in improving the health status and HRQoL of subjects with MDD. To evaluate the health status and HRQoL, the patients completed the EQ-5D-5L, which is a generic multiattribute measure of health that is broadly applied in medical settings (EQ-5D-5L, 2024; Feng et al., 2021). The tool consists of the following: 1) a descriptive system comprising five dimensions with one item per dimension, namely mobility (MO), self-care (SC), usual activities (UA), pain/discomfort (PD), and anxiety/depression (AD), which are rated on a five-point scale ranging from “no problem” to “unable to/extreme problems”; 2) a thermometer-like visual–analog scale (VAS) ranging from 0 (worst imaginable health) to 100 (best imaginable health) (Golicki and Niewada, 2017). Based on the responses to the descriptive system, the health states can be summarized and represented as index values that indicate good or bad health states (EuroQol Research Foundation, 2019). The EQ-5D-5L results were compared in groups of patients receiving trazodone XR and SSRIs at all study timepoints: at baseline and after 2, 4, 8, and 12 weeks. Additionally, health profile grids were created to visualize changes in the health status profiles and calculate the probabilities of superiority in all EQ-5D-5L subscales, with separate results for the groups treated with trazodone XR and SSRIs. The probability of superiority refers to the probability that in a randomly sampled pair of subjects, i.e., within the trazodone XR or SSRIs group, the EQ-5D-5L score at a specific timepoint will be less than the score at baseline. The value of this probability ranges from 0 to 1, with results <0.5 indicating that more patients deteriorated than improved, values at 0.5 indicating that the numbers of patients improving or deteriorating were similar or did not change, and values >0.5 indicating that more patients improved than deteriorated. Moreover, profiles for mixed changes and no problems may be described, with the former indicating improvements in some EQ-5D-5L dimensions and deterioration in others while the latter designated that subjects scored “no problems” on all EQ-5D-5L areas (Devlin et al., 2020). The EQ-5D-5L scores were calculated using the eq5d package in R software (RCoreTeam, 2022). A change of 0.08 in the EQ-5D-5L index score was considered as the minimal difference for clinical importance according to the available literature for the Polish population (Henry et al., 2020).

### 2.3 Statistical analysis

Statistical analyses were performed according to the methodology used in our previous studies (Dudek et al., 2023; Siwek et al., 2023b). Data from 160 participants were included in this analysis. The descriptive characteristics as well as baseline health statuses and HRQoL values were compared using the t-test (quantitative variables) and χ^2^ (qualitative variables) test between the groups receiving trazodone XR and SSRIs. The distribution of quantitative variables was assessed using the Shapiro–Wilk test. The qualitative variables were presented as proportions, while the quantitative variables were presented in terms of means and standard deviations.

Changes in the EQ-5D-5L scores were evaluated using a linear mixed-effects model like the mixed model for repeated measures (MMRM) through the lmer function in the lme4 package of R software (version 4.2.1) (RCoreTeam, 2022). The analyses included the timepoints of measurement (0, 2, 4, 8, and 12 weeks) as well as treatment groups (trazodone XR or SSRIs) as fixed effects and the participants as the random effects (with application of the restricted maximum likelihood (REML) metric). The effects of time, treatment, and time × treatment (interactions) on the dependent variable (EQ-5D-5L scores) were calculated. The effect size was measured as the partial-eta squared for an interaction. Between-group comparisons (trazodone XR vs SSRIs) were obtained for the estimated marginal means at each timepoint. Additional analyses were performed using the same method for all outcomes, with the duration of the previous psychiatric treatment being included as a covariate in the model. The internal consistency reliability was calculated in our pilot study (Siwek et al., 2023b).

## 3 Results

### 3.1 Group characteristics

The general descriptions of the studied groups were previously published in (Dudek et al., 2023; Siwek et al., 2023b). The groups were similar in terms of the baseline levels of health status and HRQoL with regard to the mobility, self-care, and anxiety/depression domains. At baseline, the subjects treated with trazodone XR vs those receiving SSRIs presented more problems in the usual activities and pain/discomfort dimensions, with lower overall quality of life and health status as measured using the ED-5D-5L index and ED-5D-5L VAS (Table 1).

**TABLE 1:** Quality of life and overall health status of the studied groups at baseline.

EQ-5D-5L quality of life measures	SSRIs (n = 81)	Trazodone XR (n = 79)	p
Mobility: mean (SD)	1.25 (0.582)	1.30 (0.697)	p = 0.589
Self-care: mean (SD)	1.33 (0.632)	1.5 (0.895)	p = 0.081
Usual activities: mean (SD)	2.40 (1.02)	2.84 (1.21)	**P = 0.005**
Pain/discomfort: mean (SD)	1.88 (0.812)	2.20 (1.05)	**P = 0.011**
Anxiety/depression: mean (SD)	3.26 (0.959)	3.5 (1.04)	p = 0.132
Index score	0.859 (0.096)	0.773 (0.202)	**p < 0.001**
VAS: mean (SD)	49.9 (20.1)	43.4 (20.2)	**P = 0.039**

SD, standard deviation; VAS, visual–analog scale.

### 3.2 Changes in health status and HRQoL over time

The results of the MMRM approach for the HRQoL measures are displayed in Table 2. The statistically significant effects of interactions between time and treatment group are observed for the scores of self-care, usual activities, pain/discomfort, anxiety/depression, and ED-5D-5L subscales, along with the overall quality of life and health status as measured using the ED-5D-5L index and ED-5D-5L VAS. The effect sizes of the interactions between time and treatment groups (evaluated by the partial-eta squared (η^2^) value) were small for self-care, usual activities, pain/discomfort, and anxiety/depression EQ-5D-5L dimensions; small for the health status and HRQoL assessed with the EQ-5D-5L VAS; and moderate for the overall health status and quality of life measured with the ED-5D-5L index (Table 2).

**TABLE 2:** Results of the mixed-effects model-significance levels and effect sizes (partial-eta squared) for the quality of life and health status outcomes.

	Time effect, p	Treatment effect, p	Time × treatment effect, p	Partial-eta squared for interactions (95% CI)
EQ-5D-5L quality of life measures	<0.001	0.717	0.333	<0.01 (0.00–0.02)
Mobility: mean (SD)	<0.001	0.947	**0.040**	0.02 (0.00–0.04)
Self-care: mean (SD)	<0.001	0.146	**<0.001**	0.04 (0.01–0.07)
Usual activities: mean (SD)	<0.001	0.151	**0.022**	0.02 (0.00–0.04)
Pain/discomfort: mean (SD)	<0.001	0.817	**0.045**	0.02 (0.00–0.04)
Anxiety/depression: mean (SD)	<0.001	0.085	**<0.001**	0.06 (0.02–0.09)
Index score	<0.001	0.589	**<0.001**	0.04 (0.01–0.07)

CI, confidence interval; SD, standard deviation.

The estimated marginal mean for each HRQoL measure is presented in Table 3 at each timepoint along with the corresponding *p*-values for comparisons between subjects treated with SSRIs and trazodone XR. Statistically significant differences were observed between groups receiving SSRIs and trazodone XR 1) favoring SSRIs with regard to usual activities, pain/discomfort, overall quality of life, and health status at baseline and at the second week but not at later timepoints, 2) favoring trazodone XR with regard to the health status and HRQoL measured by the EQ-5D-5L VAS at the 12th week. The overall HRQoL improvement was 0.06 from the baseline to endpoint for the SSRIs group and 0.17 for the trazodone XR group (Table 3).

**TABLE 3:** Between-group comparisons of the crude HRQoL scores at each timepoint.

EQ-5D-5L quality of life measures	Baseline emmean (95% CI)			2 weeks emmean (95% CI)			4 weeks emmean (95% CI)			8 weeks emmean (95% CI)			12 weeks emmean (95% CI)		
	SSRI	T-XR	p	SSRI	T-XR	p	SSRI	T-XR	p	SSRI	T-XR	p	SSRI	T-XR	p
Mobility: mean (SD)	1.25 (1.15–1.35)	1.29 (1.18–1.4)	0.589	1.16 (1.06–1.27)	1.21 (1.1–1.31)	0.548	1.14 (1.03–1.25)	1.14 (1.04–1.26)	0.946	1.07 (0.96–1.17)	1.16 (1.05–1.27)	0.245	1.17 (1.06–1.28)	1.09 (0.98–1.21)	0.335
Self-care: mean (SD)	1.34 (1.21–1.46)	1.50 (1.37–1.63)	0.081	1.27 (1.14–1.40)	1.27 (1.14–1.40)	0.996	1.18 (1.05–1.31)	1.17 (1.03–1.30)	0.886	1.14 (1.00–1.27)	1.11 (0.97–1.25)	0.766	1.26 (1.12–1.39)	1.11 (0.97–1.25)	0.145
Usual activities: mean (SD)	2.40 (2.18–2.61)	2.85 (2.62–3.08	**0.005**	2.00 (1.78–2.22)	2.47 (2.24–2.70)	**0.004**	1.65 (1.43–1.87)	1.86 (1.63–2.09)	0.207	1.61 (1.39–1.84)	1.62 (1.38–1.86)	0.970	1.74 (1.51–1.97)	1.51 (1.26–1.75)	0.1628
Pain/discomfort: mean (SD)	1.88 (1.70–2.06)	2.21 (2.03–2.40)	**0.011**	1.66 (1.48–1.84)	1.96 (1.78–2.15)	**0.024**	1.51 (1.33–1.70)	1.64 (1.45–1.83)	0.352	1.49 (1.30–1.68)	1.52 (1.32–1.71)	0.842	1.54 (1.35–1.73)	1.49 (1.29–1.69)	0.699
Anxiety/depression: mean (SD)	3.26 (3.03–3.49)	3.51 (3.27–3.75)	0.132	2.68 (2.44–2.92)	2.89 (2.65–3.13)	0.217	2.20 (1.96–2.44)	2.25 (2.01–2.50)	0.746	1.94 (1.70–2.18)	1.88 (1.63–2.14)	0.746	2.07 (1.82–2.31)	1.73 (1.47–1.99)	0.069
Index score	0.86 (0.83–0.88)	0.77 (0.75–0.79)	**<0.0001**	0.9 (0.88–0.93)	0.851 (0.82–0.88)	**0.006**	0.94 (0.91–0.96)	0.92 (0.9–0.95)	0.507	0.94 (0.91–0.96)	0.94 (0.92–0.97)	0.679	0.92 (0.89–0.95)	0.94 (0.92–0.98)	0.169
VAS: mean (SD)	50.0 (45.7–54.3)	43.4 (38.9–47.9)	**0.039**	57.4 (52.9–61.8)	50.5 (46.0–55.0)	**0.033**	63.2 (58.7–67.6)	60.9 (56.3–65.5)	0.485	67.2 (62.7–71.7)	68.5 (63.7–73.2)	0.704	64.9 (60.3–69.5)	72.8 (67.9–77.6)	**0.021**

CI, confidence interval; emmean, estimated marginal mean; SD, standard deviation; SSRI, group receiving selective serotonin reuptake inhibitor; T–XR, group receiving trazodone extended release formulation; VAS, visual–analog scale.

The results of the MMRM approach for the HRQoL measures considering the duration of previous psychiatric treatment as a covariate are presented in Table 4. Statistically significant effects of the interactions between time and treatment groups were observed for the scores of self-care, usual activities, pain/discomfort, anxiety/depression, and ED-5D-5L subscales as well as the overall quality of life and health status assessed using the ED-5D-5L index and ED-5D-5L VAS. The effect sizes of the interactions between time and treatment groups were moderate for the ED-5D-5L index (η^2^ = 0.06) as well as for self-care (η^2^ = 0.02), usual activities (η^2^ = 0.04), pain/discomfort (η^2^ = 0.02), anxiety/depression (η^2^ = 0.02), and ED-5D-5L VAS (η^2^ = 0.05) (Table 4).

**TABLE 4:** Results of the mixed-effects model with the duration of previous psychiatric treatment as a covariate showing the significance levels and effect sizes (partial-eta squared) for the quality of life and health status outcomes.

EQ-5D-5L quality of life measures	Treatment effect, p	Time effect, p	Time × treatment effect, p	Partial-eta squared for interactions (95% CI)
Mobility: mean (SD)	0.907	0.002	0.293	<0.001 (0.00–0.03)
Self-care: mean (SD)	0.769	<0.001	**0.029**	0.02 (0.00–0.05)
Usual activities: mean (SD)	0.126	<0.001	**<0.001**	0.04 (0.01–0.08)
Pain/discomfort: mean (SD)	0.092	<0.001	**0.029**	0.02 (0.00–0.05)
Anxiety/depression: mean (SD)	0.452	<0.001	**0.044**	0.02 (0.00–0.04)
Index score	0.057	<0.001	**<0.001**	0.06 (0.02–0.10)
VAS: mean (SD)	0.283	<0.001	**<0.001**	0.05 (0.02–0.09)

CI, confidence interval; SD, standard deviation; VAS, visual–analog scale.

**FIGURE 1:**
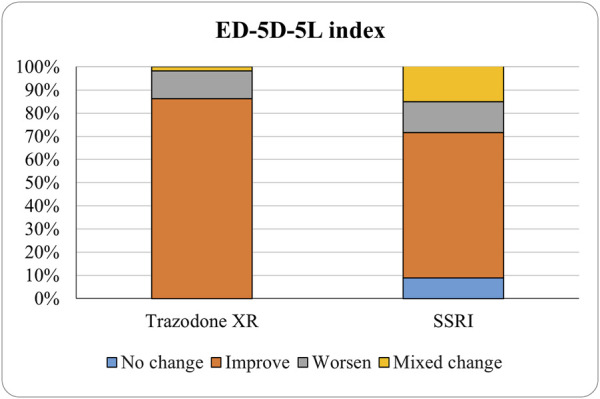
Changes in the health statuses of the studied groups from baseline to endpoint.

**FIGURE 2:**
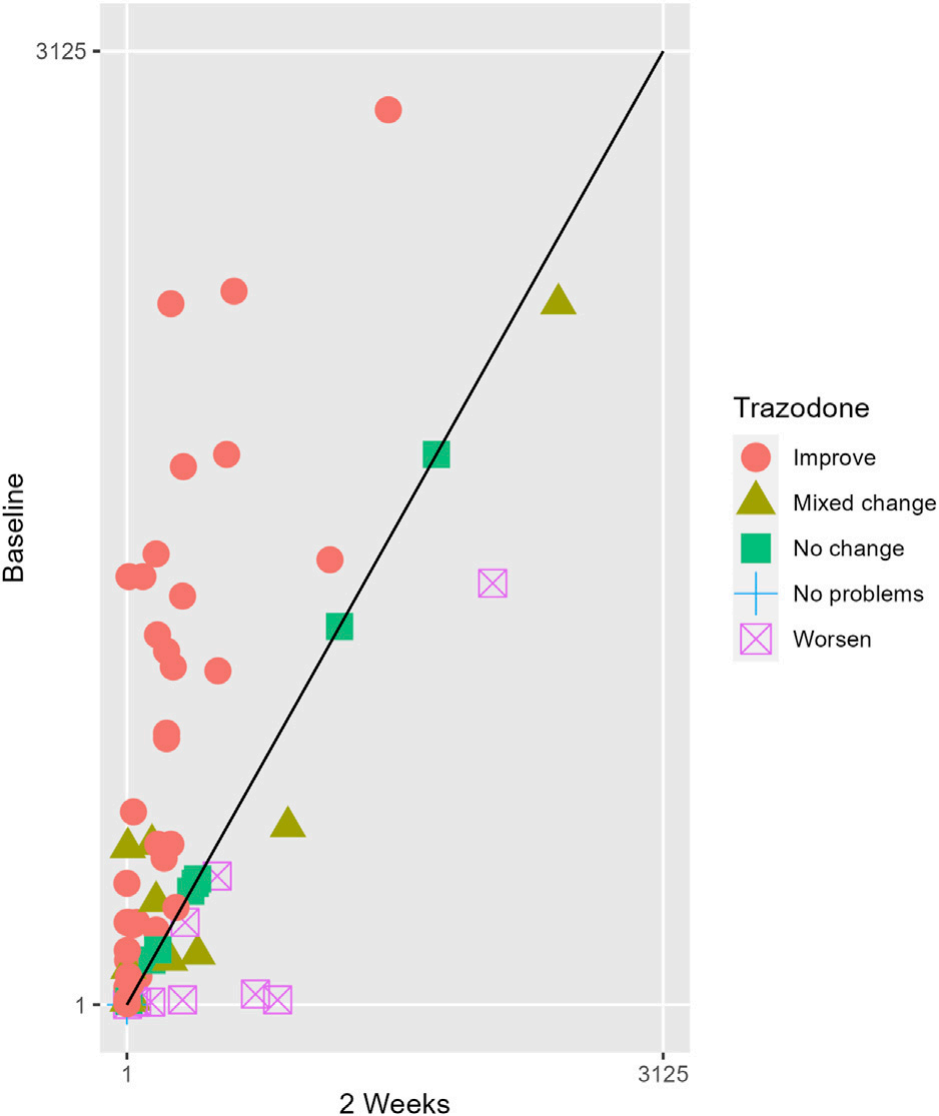
Health profile grid presenting changes in the health status of the group receiving trazodone XR at baseline vs second week of treatment.

**FIGURE 3:**
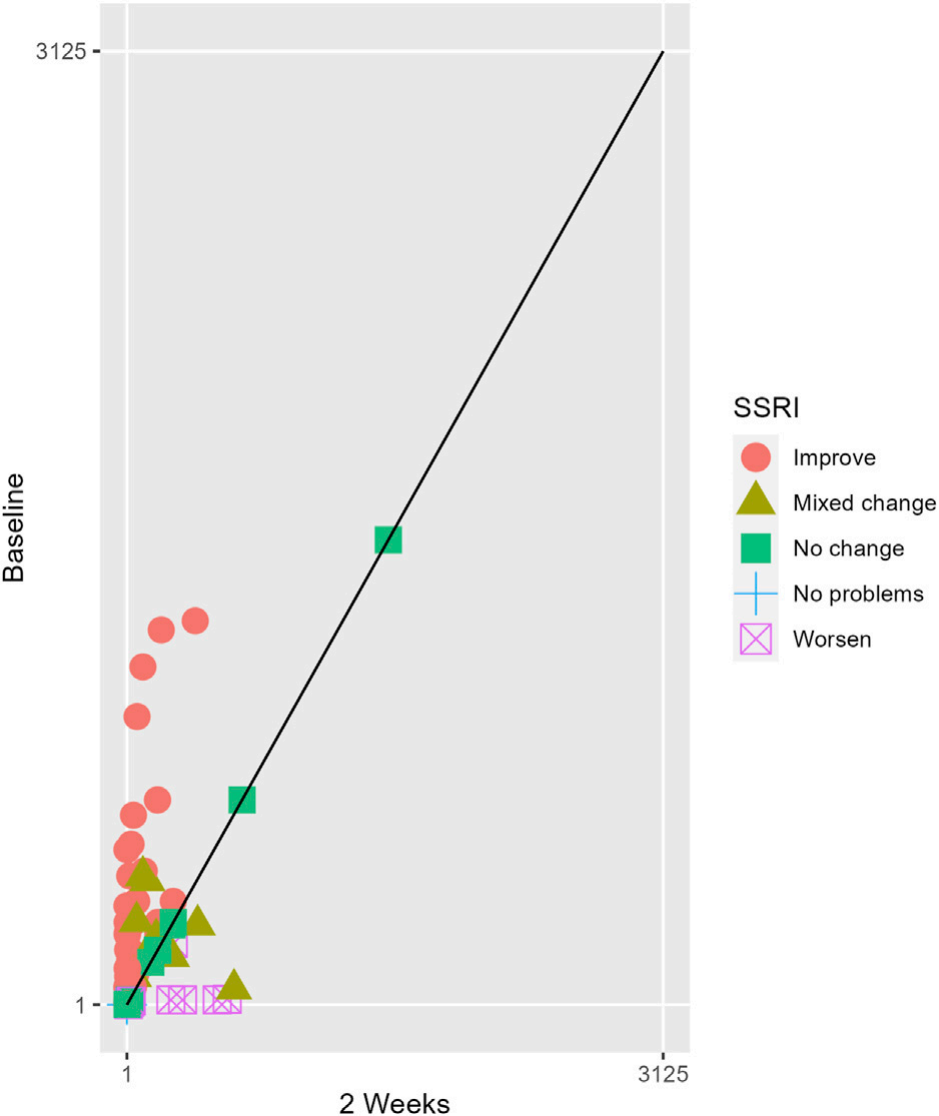
Health profile grid presenting changes in the health status of the group receiving SSRIs at baseline vs second week of treatment.

**FIGURE 4:**
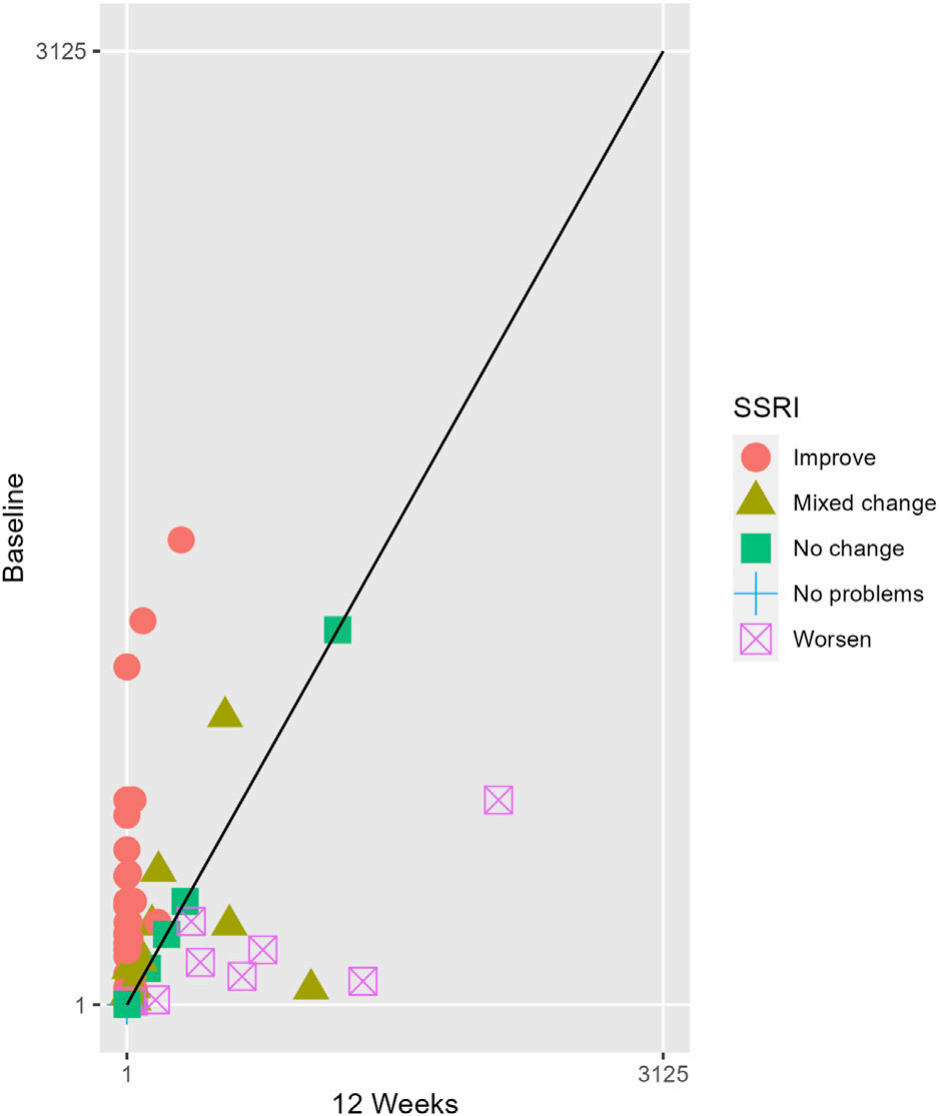
Health profile grid presenting changes in the health status of the group receiving trazodone XR at baseline vs 12th week of treatment.

**FIGURE 5:**
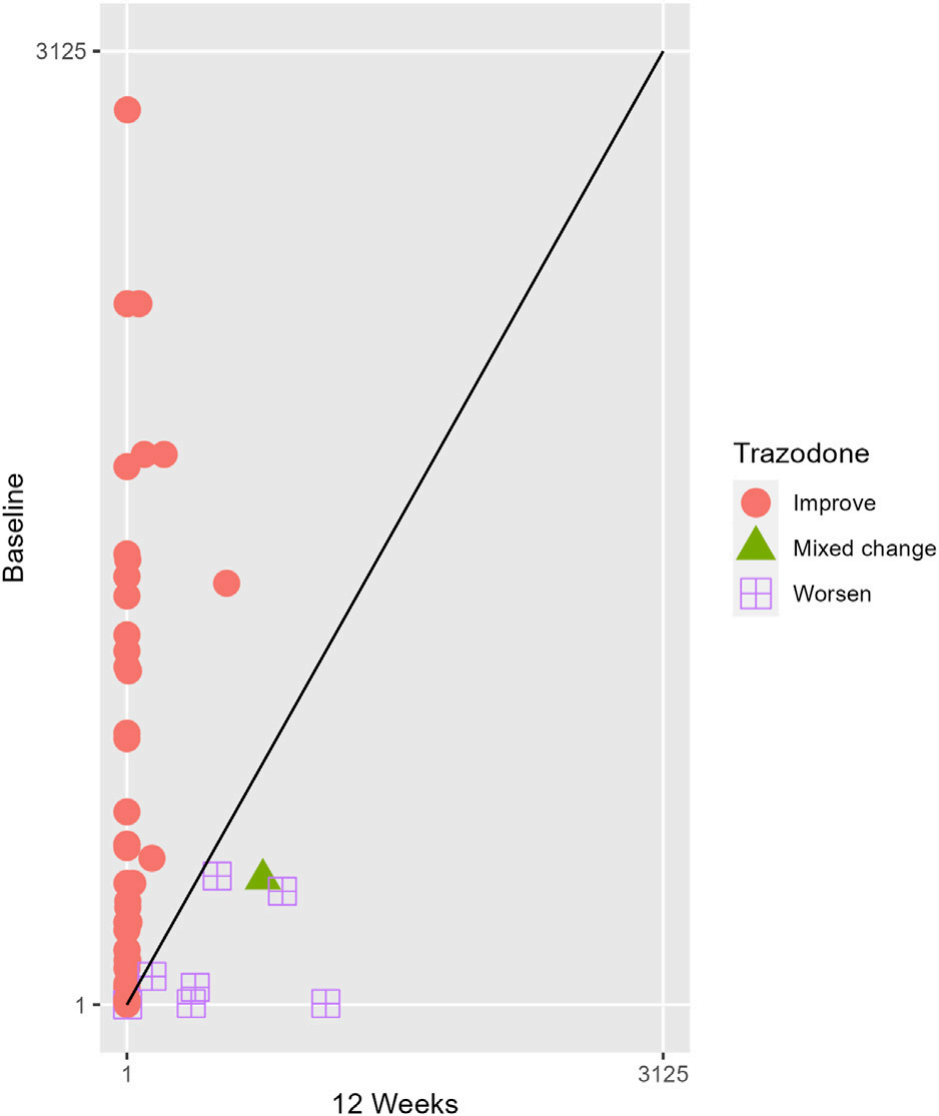
Health profile grid presenting changes in the health status of the group receiving SSRIs at baseline vs 12th week of treatment.

The proportions of subjects who presented specific health status profile changes among the subjects treated with SSRIs and trazodone XR are presented in Supplementary Table S1 along with the corresponding *p*-values for comparisons between subsequent timepoints. Statistically significant differences were observed between the groups receiving SSRIs and trazodone XR in these subjects presenting changes in the overall quality of life evaluated with EQ-5D-5L index in the comparisons from baseline to 12th week; here, more participants presented higher percentages of “improved” health status in the trazodone XR vs SSRIs group (*p* = 0.02 for post-hoc comparisons) and higher percentages of “mixed changes” in the SSRIs vs trazodone XR group (*p* = 0.02 for post-hoc comparisons) (Supplementary Table S1; Figure 1). Moreover, a observable trend (*p* = 0.05) suggested that the SSRI and trazodone XR groups varied in terms of the percentage of patients presenting changes in the usual activities subscale of EQ-5D-5L in the comparison over baseline to 12th week, but post-hoc analyses yielded no significant differences between the percentages of subjects showing “improved”, “mixed change,” or “worsened” health statuses (Supplementary Table S1). Visualizations of the changes in the health statuses of the trazodone XR and SSRIs groups from baseline to each timepoint are depicted in Figures 2–5.

**FIGURE 6:**
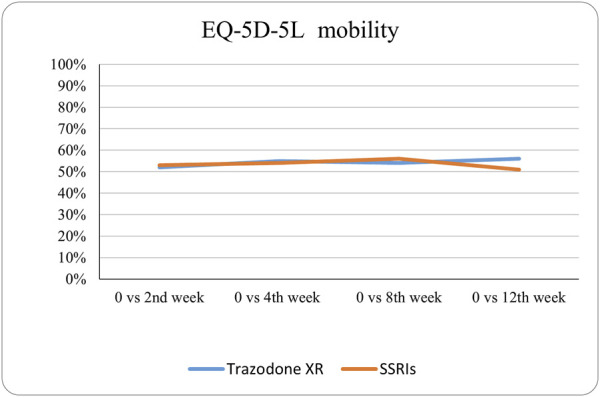
Probability of superiority of intervention at all the timepoints in the studied groups for mobility with regard to quality of life. The mobility level is presented on a scale of 0% (unable to/extreme problems) to 100% (no problem).

**FIGURE 7:**
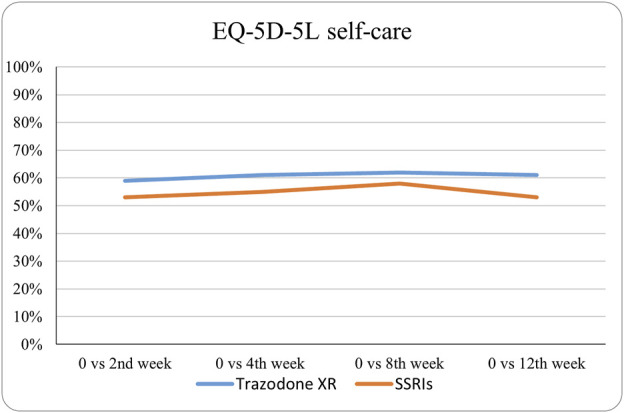
Probability of superiority of intervention at all the timepoints in the studied groups for self-care with regard to quality of life. The self-care level is presented on a scale of 0% (unable to/extreme problems) to 100% (no problem).

**FIGURE 8:**
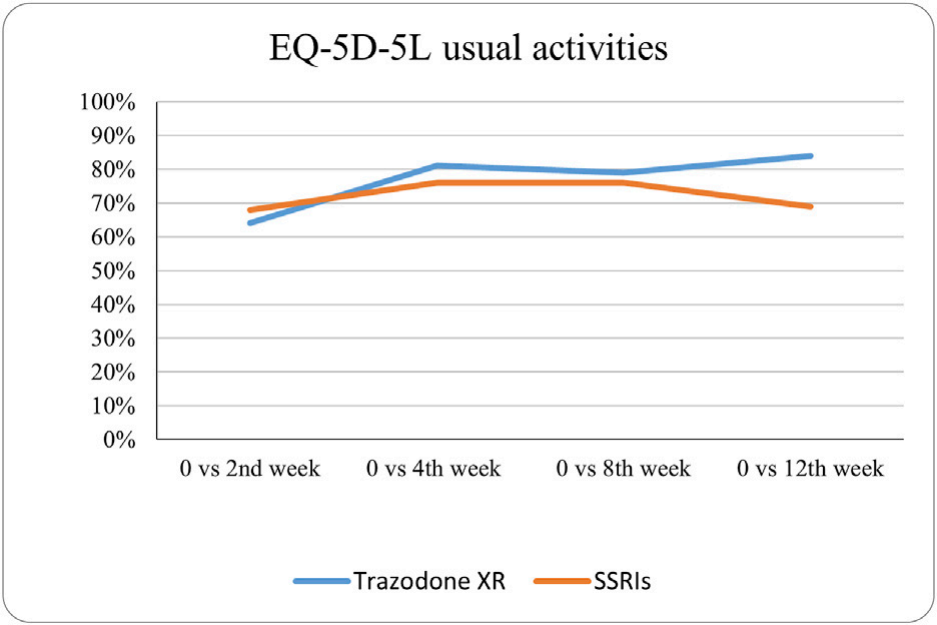
Probability of superiority of intervention at all the timepoints in the studied groups for usual activities with regard to quality of life. The level of usual activities is presented on a scale of 0% (unable to/extreme problems) to 100% (no problem).

**FIGURE 9:**
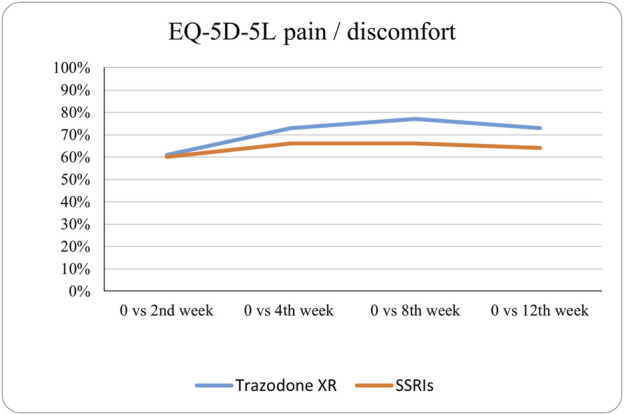
Probability of superiority of intervention at all the timepoints in the studied groups for pain/discomfort with regard to quality of life. The pain/discomfort level is presented on a scale of 0% (unable to/extreme problems) to 100% (no problem).

**FIGURE 10:**
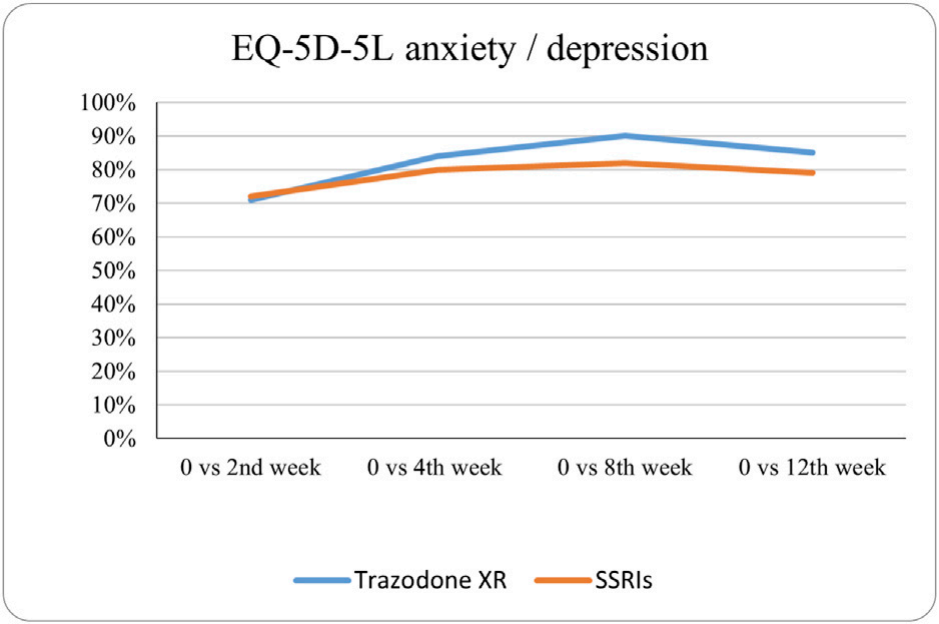
Probability of superiority of intervention at all the timepoints in the studied groups for anxiety/depression with regard to quality of life. The anxiety/depression level is presented on a scale of 0% (unable to/extreme problems) to 100% (no problem).

The probabilities of superiority for the SSRIs and trazodone XR groups are presented in Table 5 and visualized in Figures 6–10. Notably, all scores in Table 5 are higher than 0.5, meaning that more patients improved than deteriorated in both treatment groups. In the initial comparisons between baseline and the second, fourth, and eighth weeks, the scores are mixed, with some favoring SSRIs and some favoring trazodone XR, as assessed for different EQ-5D-5L dimensions; the comparisons between the baseline and 12th week favor trazodone XR over SSRIs in all dimensions of the EQ-5D-5L index (Table 5; Figures 6–10).

**TABLE 5:** Probability of superiority of the interventions at all the timepoints in the studied groups for specific domains concerning the quality of life.

EQ-5D-5L subscale	Baseline vs week 2		Baseline vs week 4		Baseline vs week 8		Baseline vs week 12	
	SSRI	T-XR	SSRI	T-XR	SSRI	T-XR	SSRI	T-XR
Mobility	0.53	0.52	0.54	0.55	0.56	0.54	0.51	0.56
Self-care	0.53	0.59	0.55	0.61	0.58	0.62	0.53	0.61
Usual activities	0.68	0.64	0.76	0.81	0.76	0.79	0.69	0.84
Pain/discomfort	0.6	0.61	0.66	0.73	0.66	0.77	0.64	0.73
Anxiety/depression	0.72	0.71	0.8	0.84	0.82	0.9	0.79	0.85

SSRI, selective serotonin reuptake inhibitor; T-XR, trazodone extended release.

## 4 Discussion

The analysis in the present study shows that trazodone XR and SSRIs are both effective in improving the health status and HRQoL during the acute phase of MDD treatment. The baseline comparisons indicate that the MDD patients treated with SSRIs and trazodone XR showed similar health status profiles and HRQoL levels in terms of the mobility, self-care, and anxiety/depression dimensions, but subjects enrolled in the trazodone XR treatment group show lower health status and HRQoL levels for usual activities and pain/discomfort along with lower overall health status and HRQoL. The initial differences may be attributed to the study design as the study was a non-randomized, open-label observation as earlier reported (Dudek et al., 2023; Siwek et al., 2023b). These differences between the treatment groups for the HRQoL were ameliorated with time, and the majority of HRQoL assessments after the 12th week of treatment indicated that trazodone XR improved the health status and HRQoL scores, which were comparable to the SSRIs pharmacotherapy; in the case of the EQ-5D-5L index, the scores were superior to those obtained for the SSRI treatment group.

The MMRM approach shows that trazodone XR is superior to SSRIs in improving several HRQoL dimensions: self-care, usual activities, pain/discomfort, anxiety/depression, as well as the overall HRQoL and health status. These results remain significant after controlling the interactions for the duration of the previous psychiatric treatment (covariate). The analysis of changes in HRQoL from baseline to the endpoint of the study indicates that significantly more subjects achieved improved health status profiles in the trazodone XR vs SSRIs group. On the other hand, significantly more participants reported mixed changes in their health status (meaning that some domains improved while the others worsened) in the SSRIs vs trazodone XR group. Moreover, the improvement in HRQoL exceeded the minimal level of clinically important difference in the trazodone XR group but not in the SSRI group. These results need to be interpreted with caution because the subjects in the trazodone XR group presented with lower initial HRQoL scores than those in the SSRI group, and it has been reported that the minimal clinically important difference may vary depending on the baseline HRQoL level (Cheng et al., 2024).

The probability of superiority analysis allowed two significant conclusions. First, both SSRI and trazodone XR treatments increase the probabilities of improved health states for all subsequent timepoints vs the baseline. Second, the probability of superiority scores evolved with different trajectories for various domains of the health status and HRQoL at subsequent timepoints vs the baseline comparisons; however, the comparison of baseline vs endpoint scores indicate higher probabilities of improved health status profiles for all dimensions in the group treated with trazodone XR vs SSRIs.

The data regarding the effectiveness of trazodone XR in improving the outcomes related to functioning, health status, and quality of life are scant. Shrashimirova et al. (2023) reported the results of a 24-week open-label study of trazodone XR treatment for MDD. Similar to our earlier results (Siwek et al., 2023b), they noted improved overall functioning as well as work/school, social life, and family life/domestic responsibilities when assessed with the SDS at subsequent timepoints (12th, 18^th^, and 24th weeks). Moreover, these authors reported that trazodone XR treatment resulted in health status and HRQoL improvements when assessed by EQ-5D-5L at subsequent timepoints, but they did not elaborate on these results. Tellone et al. (2024) reported the results of an 8-week observation study of trazodone XR vs SSRIs in MDD. Although they showed that trazodone XR offered slightly better improvement of HRQoL than SSRIs, they used a different tool to measure the HRQoL (i.e., quality-of-life enjoyment and satisfaction questionnaire short form) and therefore could not perform a more thorough analysis of the impacts of trazodone XR and SSRIs on specific dimensions of HRQoL (Tellone et al., 2024). The results presented in our work expand current knowledge on the impact of trazodone XR has on the functioning of depressed subjects and offers several new insights: 1) trazodone XR is superior to SSRIs in improving some dimensions of health status and HRQoL, such as self-care, usual activities, pain/discomfort, and anxiety/depression along with the overall health status and HRQoL. Interestingly, in contrast to the results reported by Tellone et al. (2024) who found that trazodone XR offered a somewhat better improvement in HRQoL than SSRIs after 8 weeks, the differences in the impacts of trazodone XR and SSRIs on the participants health status profiles and HRQoL in our study were significant only after 12 weeks of treatment. This may be attributed to the fact that the participants in the trazodone XR group in our sample study presented with lower initial HRQoL and health statuses than those in the SSRI group. Notably, our previous study suggested that trazodone XR was more effective in reducing the severity of depression, anxiety, and insomnia as well as achieving treatment responses vs SSRIs (Dudek et al., 2023); therefore, it is understandable that it offered favorable outcomes for the health status and HRQoL, as it was demonstrated earlier that symptomatic improvement is significantly linked to normalization of HRQoL (Steiner et al., 2017). Furthermore, some of the differences in HRQoL between trazodone XR and SSRIs could be due to the dissimilar pharmacodynamic profiles of these drugs as well as multimodal characteristics of trazodone XR (Fagiolini et al., 2023) and/or the adverse effects of SSRIs, such as emotional blunting (Fagiolini et al., 2021), sexual dysfunction (Jing and Straw-Wilson, 2016), and sleep disruptions (Wichniak et al., 2017), which are less common (or better yet improved) during trazodone XR treatment (Dudek et al., 2023; Siwek et al., 2023a; Siwek et al., 2023b).

Several limitations of this work need to be acknowledged, such as the open-label design, lack of randomization that could have led to dissimilarities between the treatment groups at baseline, study being conducted at a single center, categorization of SSRIs as an entire group, and flexible dosing of the antidepressant drugs. Therefore, our results need corroboration through further studies with a more rigid methodology. Nevertheless, these factors do not undermine the value of this work, which lies in the impacts of trazodone XR and SSRIs treatments on the quality of life of MDD subjects. The main strength of this work lies in its’ “real-life” design of the study where the majority of outcomes are rated by patients rather than clinicians, which was intended to achieve patient-centered results while focusing on the aspects of MDD pharmacotherapy that are most important for MDD subjects (Zimmerman et al., 2006; Zimmerman and Lin, 2023). Another advantage of this study is the use of the EQ-5D-5L tool, which allows feasible assessment of the HRQoL and provides translatable data for comparisons across different diagnostic categories and medical fields, between patients and general populations, and against locally specific population norms (Devlin et al., 2022). Although the number of MDD studies incorporating the EQ-5D-5L assessment is very low, it has been used in two significant projects, i.e., RCTs assessing the efficacies of esketamine nasal spray adjunctive to standard of care treatment in MDD patients with suicidal ideation with intent (Jamieson et al., 2023a) and in treatment-resistant depression (Jamieson et al., 2023b); these RCTs are large observation studies evaluating the evolution of functional outcomes in MDD participants receiving pharmacotherapy over a duration of 6 months (Noto et al., 2022) and explore the risk of relapse in stable MDD participants who maintained pharmacotherapy or withdrew from it (Duffy et al., 2019). While (Brockbank et al., 2021) recommended the use of EQ-5D tools in trials which precede reimbursement submissions to the health technology agencies, we believe that the EQ-5D tool should be implemented in “real-life” trials of already marketed antidepressants to inform clinical practice while choosing appropriate treatment drugs for MDD patients.

## 5 Conclusion

In summary, the present study was among the few works that provide direct comparisons of the impacts of trazodone XR and SSRIs on the health status and HRQoL based on naturalistic observations. The results show that treatments with both trazodone XR and SSRIs result in significant improvements of the health status and HRQoL and that the patients treated with trazodone XR achieved improved health status profiles more often than those receiving SSRIs. The findings of this work are in agreement with our previous results, indicating that trazodone XR should be considered as one of the first-line antidepressant options in the treatment of MDD.

## Data Availability

The raw data supporting the conclusions of this article will be made available by the authors, without undue reservation.

## References

[B1] Albert U. Tomasetti C. Marra C. Neviani F. Pirani A. Taddeo D. (2023). Treating depression in clinical practice: new insights on the multidisciplinary use of trazodone. Front. Psychiatry 14, 1207621. 10.3389/fpsyt.2023.1207621 37654988 PMC10466041

[B2] Bortolato B. Miskowiak K. W. Köhler C. A. Maes M. Fernandes B. S. Berk M. (2016). Cognitive remission: a novel objective for the treatment of major depression? BMC Med. 14, 9–18. 10.1186/s12916-016-0560-3 26801406 PMC4724131

[B3] Brockbank J. Krause T. Moss E. Pedersen A. M. Mørup M. F. Ahdesmäki O. (2021). Health state utility values in major depressive disorder treated with pharmacological interventions: a systematic literature review. Health Qual. Life Outcomes 19, 94–17. 10.1186/s12955-021-01723-x 33736649 PMC7977292

[B4] Cao B. Park C. Subramaniapillai M. Lee Y. Iacobucci M. Mansur R. B. (2019). The efficacy of vortioxetine on anhedonia in patients with major depressive disorder. Front. Psychiatry 10, 17–18. 10.3389/fpsyt.2019.00017 30766492 PMC6365446

[B5] Cao B. Xu L. Chen Y. Wang D. Lee Y. Rosenblat J. D. (2022). Comparative efficacy of pharmacological treatments on measures of self-rated functional outcomes using the Sheehan Disability Scale in patients with major depressive disorder: a systematic review and network meta-analysis. CNS Spectrums 27, 1–9. 10.1017/S1092852921000171 33583460

[B6] Cheng L. J. Chen L. A. Cheng J. Y. Herdman M. Luo N. (2024). Systematic review reveals that EQ-5D minimally important differences vary with treatment type and may decrease with increasing baseline score. J. Clin. Epidemiol. 174, 111487. 10.1016/j.jclinepi.2024.111487 39084578

[B7] Chokka P. Bougie J. Proulx J. Tvistholm A. H. Ettrup A. (2019). Long-term functioning outcomes are predicted by cognitive symptoms in working patients with major depressive disorder treated with vortioxetine: results from the AtWoRC study. CNS Spectrums 24, 616–627. 10.1017/S1092852919000786 30802419

[B8] Cipriani A. Furukawa T. A. Salanti G. Chaimani A. Atkinson L. Z. Ogawa Y. (2018). Comparative efficacy and acceptability of 21 antidepressant drugs for the acute treatment of adults with major depressive disorder: a systematic review and network meta-analysis. Lancet 391, 1357–1366. 10.1016/S0140-6736(17)32802-7 29477251 PMC5889788

[B9] Crapanzano C. Casolaro I. Krupa A. J. (2024). Clinical experience with parenteral trazodone in mood disorders: a literature review. Psychiatr. Pol. 58, 449–466. 10.12740/PP/182933 39217422

[B10] de Beste L. Ossendrijver I. Koet L. van Tilborg-Den Boeft M. (2024). Depression follow-up monitoring with the PHQ-9: open cluster-randomised controlled trial. Br. J. General Pract. 74, 202–203. 10.3399/bjgp24X737109 PMC11060819 38664056

[B11] Dennehy E. B. Marangell L. B. Martinez J. Balasubramani G. K. Wisniewski S. R. (2014). Clinical and functional outcomes of patients who experience partial response to citalopram: secondary analysis of STAR*D. J. Psychiatric Pract. 20, 178–187. 10.1097/01.pra.0000450317.76117.62 24847991

[B12] Devlin N. Parkin D. Janssen B. (2020). Methods for analysing and reporting EQ-5D data. 10.1007/978-3-030-47622-9 33347096

[B13] Devlin N. Pickard S. Busschbach J. (2022). “The development of the EQ-5D-5L and its value sets,” in Value sets for EQ-5D-5L: a compendium, comparative review and user guide, 1–12. 10.1007/978-3-030-89289-0_1

[B14] Dudek D. Chrobak A. A. Krupa A. J. Gorostowicz A. Gerlich A. Juryk A. (2023). TED—trazodone effectiveness in depression: a naturalistic study of the effeciveness of trazodone in extended release formulation compared to SSRIs in patients with a major depressive disorder. Front. Pharmacol. 14, 1296639. 10.3389/fphar.2023.1296639 38027034 PMC10646491

[B15] Duffy L. Bacon F. Clarke C. S. Donkor Y. Freemantle N. Gilbody S. (2019). A randomised controlled trial assessing the use of citalopram, sertraline, fluoxetine and mirtazapine in preventing relapse in primary care patients who are taking long-term maintenance antidepressants ANTLER: ANTidepressants to prevent reLapse in dEpRes. Trials 20, 1–13. 10.1186/s13063-019-3390-8 31159856 PMC6547591

[B16] EQ-5D-5L (2024). Information and support. Euroqol-instruments. Available at: https://euroqol.org/information-and-support/euroqol-instruments/eq-5d-5l/.

[B17] Fagiolini A. Florea I. Loft H. Christensen M. C. (2021). Effectiveness of vortioxetine on emotional blunting in patients with major depressive disorder with inadequate response to SSRI/SNRI treatment. J. Affect. Disord. 283, 472–479. 10.1016/j.jad.2020.11.106 33516560

[B18] Fagiolini A. González-Pinto A. Miskowiak K. W. Morgado P. Young A. H. Vieta E. (2023). Role of trazodone in treatment of major depressive disorder: an update. Ann. General Psychiatry 22, 32. 10.1186/s12991-023-00465-y PMC10474647 37660092

[B19] Feng Y. S. Kohlmann T. Janssen M. F. Buchholz I. (2021). Psychometric properties of the EQ-5D-5L: a systematic review of the literature. Qual. Life Res. 30, 647–673. 10.1007/s11136-020-02688-y 33284428 PMC7952346

[B20] Golicki D. Niewada M. (2017). EQ-5D-5L Polish population norms. Archives Med. Sci. 13, 191–200. 10.5114/aoms.2015.52126 PMC5206353 28144271

[B21] Henry E. B. Barry L. E. Hobbins A. P. McClure N. S. O’Neill C. (2020). Estimation of an instrument-defined minimally important difference in EQ-5D-5L index scores based on scoring algorithms derived using the EQ-VT version 2 valuation protocols. Value Health 23, 936–944. 10.1016/j.jval.2020.03.003 32762996

[B22] Jamieson C. Canuso C. M. Ionescu D. F. Lane R. Qiu X. Rozjabek H. (2023a). Effects of esketamine on patient-reported outcomes in major depressive disorder with active suicidal ideation and intent: a pooled analysis of two randomized phase 3 trials (ASPIRE I and ASPIRE II). Qual. Life Res. 32, 3053–3061. 10.1007/s11136-023-03451-9 37439961 PMC10522733

[B23] Jamieson C. Popova V. Daly E. Cooper K. Drevets W. C. Rozjabek H. M. (2023b). Assessment of health-related quality of life and health status in patients with treatment-resistant depression treated with esketamine nasal spray plus an oral antidepressant. Health Qual. Life Outcomes 21, 40–49. 10.1186/s12955-023-02113-1 37158911 PMC10169482

[B24] Jing E. Straw-Wilson K. (2016). Sexual dysfunction in selective serotonin reuptake inhibitors (SSRIs) and potential solutions: a narrative literature review. Ment. Health Clin. 6, 191–196. 10.9740/mhc.2016.07.191 29955469 PMC6007725

[B25] Kennedy S. H. (2022). Beyond response: aiming for quality remission in depression. Adv. Ther. 39, 20–28. 10.1007/s12325-021-02030-z 35247185 PMC9015986

[B26] Kikuchi T. Iga J. I Oosawa M. Hoshino T. Moriguchi Y. Izutsu M. (2024). A web-based survey on the occurrence of emotional blunting in patients with major depressive disorder in Japan: patient perceptions and attitudes. Neuropsychopharmacol. Rep. 44, 321–332. 10.1002/npr2.12417 38616339 PMC11144621

[B27] Kowalczyk E. Borysowski J. Ordak M. Kniotek M. Radziwoń-Zaleska M. Siwek M. (2024). Placebo-controlled randomized clinical trials of antidepressants for major depressive disorder: analysis of ClinicalTrials.gov, 2008–2022. Psychiatry Res. 333, 115730. 10.1016/j.psychres.2024.115730 38245978

[B28] McIntyre R. S. Lee Y. Mansur R. B. (2015). Treating to target in major depressive disorder: response to remission to functional recovery. CNS Spectrums 20, 20–30. 10.1017/S1092852915000826 26683526

[B29] Miller I. W. Keitner G. I. Schatzberg A. F. Klein D. N. Thase M. E. Rush A. J. (1998). The treatment of chronic depression, part 3: psychosocial functioning before and after treatment with sertraline or imipramine. J. Clin. Psychiatry 59, 608–619. 10.4088/JCP.v59n1108 9862607

[B30] Noto S. Wake M. Mishiro I. Hammer-Helmich L. Ren H. Moriguchi Y. (2022). Health-related quality of life over 6 Months in patients with major depressive disorder who started antidepressant monotherapy. Value Health Regional Issues 30, 127–133. 10.1016/j.vhri.2021.12.001 35405582

[B31] RCoreTeam (2022). “R: a language and environment for statistical computing,” in Vienna: R foundation for statistical computing. R: a language and environment for statistical computing. Vienna, Austria: (R Foundation for Statistical Computing).

[B32] EuroQol Research Foundation (2019). EQ-5D: EQ-5D-5L user guide. Available at: https://euroqol.org/publications/user-guides .

[B33] Rodolico A. Cutrufelli P. Di Francesco A. Aguglia A. Catania G. Concerto C. (2024). Efficacy and safety of ketamine and esketamine for unipolar and bipolar depression: an overview of systematic reviews with meta-analysis. Front. Psychiatry 15, 1325399. 10.3389/fpsyt.2024.1325399 38362031 PMC10867194

[B34] Sheehan D. V. Nakagome K. Asami Y. Pappadopulos E. A. Boucher M. (2017). Restoring function in major depressive disorder: a systematic review. J. Affect. Disord. 215, 299–313. 10.1016/j.jad.2017.02.029 28364701

[B43] Shrashimirova M. Tyanev I. Cubała W. J. Wichniak A. Vodickova-Borzova C. Ruggieri A.(2023). Long-term treatment with trazodone once-a-day (TzOAD) in patients with MDD: an observational, prospective study. Neuropsychiatr. Dis. Treat. 19, 1181–1193. 10.2147/NDT.S399948 37201102 PMC10187683

[B35] Siwek M. Chrobak A. A. Krupa A. J. Gorostowicz A. Gerlich A. Juryk A. (2023a). TED (Trazodone Effectiveness in Depression): effectiveness of trazodone extended-release in subjects with unsatisfactory response to SSRIs. Psychiatr. Pol. 2674, 1–18. 10.12740/pp/onlinefirst/174432 38484384

[B36] Siwek M. Gorostowicz A. Chrobak A. A. Gerlich A. Krupa A. J. Juryk A. (2023b). TED—trazodone efficacy in depression: a naturalistic study on the efficacy of trazodone in an extended-release formulation compared to SSRIs in patients with a depressive episode—preliminary report. Brain Sci. 13, 86. 10.3390/brainsci13010086 36672067 PMC9856641

[B37] Soares C. N. Wajsbrot D. B. Boucher M. (2020). Predictors of functional response and remission with desvenlafaxine 50 mg and 100 mg: a pooled analysis of randomized, placebo-controlled studies in patients with major depressive disorder. CNS Spectrums 25, 363–371. 10.1017/S1092852919000828 31060632

[B38] Steiner A. J. Recacho J. Vanle B. Dang J. Wright S. M. Miller J. S. (2017). Quality of life, functioning, and depressive symptom severity in older adults with major depressive disorder treated with citalopram in the STAR*D study. J. Clin. Psychiatry 78, 897–903. 10.4088/JCP.16m11335 28858443

[B39] Tellone V. Markovic O. Strashimirova M. Sani G. Lenderking W. R. Margolis M. K. (2024). Impact of trazodone once-a-day on quality of life and functional recovery in adults with major depressive disorder: a prospective, observational study. Brain Behav. 14, e3610. 10.1002/brb3.3580 39034363 PMC11260556

[B40] Wichniak A. Wierzbicka A. Walęcka M. Jernajczyk W. (2017). Effects of antidepressants on sleep. Curr. Psychiatry Rep. 19, 63–67. 10.1007/s11920-017-0816-4 28791566 PMC5548844

[B41] Zimmerman M. Lin S. Y. (2023). 50% improvement: should treatment response go beyond symptom improvement when evaluating the treatment of depression? J. Clin. Psychiatry 84, 22m14706. 10.4088/JCP.22m14706 37167567

[B42] Zimmerman M. McGlinchey J. B. Posternak M. A. Friedman M. Attiullah N. Boerescu D. (2006). How should remission from depression Be defined? The depressed patient’s perspective. Am. J. Psychiatry 163, 148–150. 10.1176/appi.ajp.163.1.148 16390903

