# Supplementary material for: Trazodone effectiveness in depression: impacts of trazodone extended release vs SSRIs on the health status and quality of life of patients with major depressive disorder
Source: Front Pharmacol. 2025 Jan 23;15:1525498. doi: 10.3389/fphar.2024.1525498 (PMC11798982; doi:10.3389/fphar.2024.1525498)
Supplement: Supplementary file 1 [file Table1.docx]

Table S1. Changes in health-status profiles of the studied groups for each timepoint

| Health status | **Baseline vs. 2 weeks** | | | **Baseline vs. 4 weeks** | | | **Baseline vs. 8 weeks** | | | **Baseline vs. 12 weeks** | | |
| --- | --- | --- | --- | --- | --- | --- | --- | --- | --- | --- | --- | --- |
|  | **ED-5D-5L index** | | | | | | | | | | | |
|  | **SSRI** | **T-XR** | **Chi2** | **SSRI** | **T-XR** | **Chi2** | **SSRI** | **T-XR** | **Chi2** | **SSRI** | **T-XR** | **Chi2** |
| **No change (%)** | 16.4 | 13 | no change, improve, worsen: ꭓ2= 1.055, df= 3, p= 0.79  total vs no problems:  ꭓ2= 0.002, df= 1, p= 0.96 | 8.2 | 4.7 | no change, improve, worsen: ꭓ2= 1.55, df= 2, p= 0.46  total vs no problems:  ꭓ2= 0.009, df= 1, p= 0.92 | 2.8 | 1.7 | no change, improve, worsen: ꭓ2= 1.785, df= 2, p= 0.4  total vs no problems:  ꭓ2= 0.017, df= 1, p= 0.89 | 8.9 | 0 | no change, improve, worsen: ꭓ2= 13.7, df= 3, **p= 0.003**  total vs no problems:  ꭓ2= 0.86, df= 1, p= 0.35 |
| **Improve (%)** | 61.6 | 58 |  | 64.4 | 79.7 |  | 74.6 | 83.1 |  | 62.7 | 86.2 |  |
| **Worsen (%)** | 11 | 14.5 |  | 8.2 | 6.2 |  | 8.5 | 3.4 |  | 13.4 | 12.1 |  |
| **Mixed change (%)** | 11 | 14.5 |  | 19.2 | 9.4 |  | 14.1 | 11.9 |  | 16.4 | 1.7 |  |
| **Total with problems (%)** | 98.6 | 98.6 |  | 98.6 | 98.5 |  | 98.6 | 98.3 |  | 98.5 | 100 |  |
| **No problems (%)** | 1.4 | 1.4 |  | 1.4 | 1.5 |  | 1.4 | 1.7 |  | 1.5 | 0 |  |
| Health status | **Baseline vs. 2 weeks** | | | **Baseline vs. 4 weeks** | | | **Baseline vs. 8 weeks** | | | **Baseline vs. 12 weeks** | | |
|  | **ED-5D-5L mobility** | | | | | | | | | | | |
|  | **SSRI** | **T-XR** | **Chi2** | **SSRI** | **T-XR** | **Chi2** | **SSRI** | **T-XR** | **Chi2** | **SSRI** | **T-XR** | **Chi2** |
| **No change (%)** | 58.3 | 31.2 | no change, improve, worsen: ꭓ2= 4.18, df = 2, p= 0.12  total vs no problems:  ꭓ2= 1.01, df= 1, p= 0.31 | 23.1 | 28.6 | no change, improve, worsen: ꭓ2= 1.06, df= 2, p= 0.95  total vs no problems:  ꭓ2= 0.349, df= 1, p= 0.55 | 23.1 | 15.4 | no change, improve, worsen: ꭓ2= 1.259, df= 2, p= 0.53  total vs no problems:  ꭓ2= 0.27, df= 1, p= 0.6 | 20 | 8.3 | no change, improve, worsen: ꭓ2= 2.23, df= 2, p= 0.33  total vs no problems:  ꭓ2= 0.035, df= 1, p= 0.85 |
| **Improve (%)** | 41.7 | 43.8 |  | 61.5 | 57.1 |  | 69.2 | 61.5 |  | 46.7 | 75 |  |
| **Worsen (%)** | 0 | 25 |  | 15.4 | 14.3 |  | 7.7 | 23.1 |  | 33.3 | 16.7 |  |
| **Mixed change (%)** | 16.2 | 22.9 |  | 17.6 | 21.5 |  | 18.1 | 21.7 |  | 22.1 | 20.7 |  |
| **Total with problems (%)** | 83.8 | 77.1 |  | 82.4 | 78.5 |  | 81.9 | 78.3 |  | 77.9 | 79.3 |  |
| **No problems (%)** | 58.3 | 31.2 |  | 23.1 | 28.6 |  | 23.1 | 15.4 |  | 20 | 8.3 |  |
| Health status | **Baseline vs. 2 weeks** | | | **Baseline vs. 4 weeks** | | | **Baseline vs. 8 weeks** | | | **Baseline vs. 12 weeks** | | |
|  | **ED-5D-5L self-care** | | | | | | | | | | | |
|  | **SSRI** | **T-XR** | **Chi2** | **SSRI** | **T-XR** | **Chi2** | **SSRI** | **T-XR** | **Chi2** | **SSRI** | **T-XR** | **Chi2** |
| **No change (%)** | 38.1 | 30 | no change, improve, worsen: ꭓ2= 2.79, df = 2, p= 0.25  total vs no problems:  ꭓ2= 0.001, df= 1, p= 0.97 | 20 | 15.8 | no change, improve, worsen: ꭓ2= 2.252, df= 2, p= 0.32  total vs no problems:  ꭓ2= 0.082, df= 1, p= 0.77 | 10.5 | 11.8 | no change, improve, worsen: ꭓ2= 2.932, df= 2, p= 0.23  total vs no problems:  ꭓ2= 0.062, df= 1, p= 0.8 | 20 | 10.5 | no change, improve, worsen: ꭓ2= 3.364, df= 2, p= 0.16  total vs no problems:  ꭓ2= 0.164, df= 1, p= 0.69 |
| **Improve (%)** | 42.9 | 65 |  | 60 | 78.9 |  | 73.7 | 88.2 |  | 50 | 78.9 |  |
| **Worsen (%)** | 19 | 5 |  | 20 | 5.3 |  | 15.8 | 0 |  | 30 | 10.5 |  |
| **Mixed change (%)** | 28.4 | 28.6 |  | 27 | 29.2 |  | 26.4 | 28.3 |  | 29.4 | 32.8 |  |
| **Total with problems (%)** | 71.6 | 71.4 |  | 73 | 70.8 |  | 73.6 | 71.7 |  | 70.6 | 67.2 |  |
| **No problems (%)** | 38.1 | 30 |  | 20 | 15.8 |  | 10.5 | 11.8 |  | 20 | 10.5 |  |
| Health status | **Baseline vs. 2 weeks** | | | **Baseline vs. 4 weeks** | | | **Baseline vs. 8 weeks** | | | **Baseline vs. 12 weeks** | | |
|  | **ED-5D-5L usual activities** | | | | | | | | | | | |
|  | **SSRI** | **T-XR** | **Chi2** | **SSRI** | **T-XR** | **Chi2** | **SSRI** | **T-XR** | **Chi2** | **SSRI** | **T-XR** | **Chi2** |
| **No change (%)** | 29.5 | 49.2 | no change, improve, worsen: ꭓ2= 4.95, df = 2, p= 0.08  total vs no problems:  ꭓ2= 0.617, df= 1, p= 0.43 | 14.8 | 14.3 | no change, improve, worsen: ꭓ2= 0.676, df= 2, p= 0.71  total vs no problems:  ꭓ2= 0.36, df= 1, p= 0.55 | 10 | 11.3 | no change, improve, worsen: ꭓ2= 0.14, df= 2, p= 0.93  total vs no problems:  ꭓ2= 0.664, df= 1, p= 0.42 | 17.2 | 7.8 | no change, improve, worsen: ꭓ2= 5.863, df= 2, **p= 0.05**  total vs no problems:  ꭓ2= 0.186, df= 1, p= 0.67 |
| **Improve (%)** | 57.4 | 41 |  | 73.8 | 78.6 |  | 76.7 | 77.4 |  | 63.8 | 84.3 |  |
| **Worsen (%)** | 13.1 | 9.8 |  | 11.5 | 7.1 |  | 13.3 | 11.3 |  | 19 | 7.8 |  |
| **Mixed change (%)** | 82.4 | 87.1 |  | 82.4 | 86.2 |  | 83.3 | 88.3 |  | 85.3 | 87.9 |  |
| **Total with problems (%)** | 17.6 | 12.9 |  | 17.6 | 13.8 |  | 16.7 | 11.7 |  | 14.7 | 12.1 |  |
| **No problems (%)** | 29.5 | 49.2 |  | 14.8 | 14.3 |  | 10 | 11.3 |  | 17.2 | 7.8 |  |
| Health status | **Baseline vs. 2 weeks** | | | **Baseline vs. 4 weeks** | | | **Baseline vs. 8 weeks** | | | **Baseline vs. 12 weeks** | | |
|  | **ED-5D-5L pain / discomfort** | | | | | | | | | | | |
|  | **SSRI** | **T-XR** | **Chi2** | **SSRI** | **T-XR** | **Chi2** | **SSRI** | **T-XR** | **Chi2** | **SSRI** | **T-XR** | **Chi2** |
| **No change (%)** | 30.5 | 18 | no change, improve, worsen: ꭓ2= 1.211, df = 2, p= 0.55  total vs no problems:  ꭓ2= 4.71, df= 1, p= 0.03 | 24.5 | 20 | no change, improve, worsen: ꭓ2= 1.132, df= 2, p= 0.57  total vs no problems:  ꭓ2= 0.507, df= 1, p= 0.48 | 27.1 | 15.6 | no change, improve, worsen: ꭓ2= 3.269, df= 2, p= 0.2  total vs no problems:  ꭓ2= 1.092, df= 1, p= 0.29 | 23.9 | 13.3 | no change, improve, worsen: ꭓ2= 2.346, df= 2, p= 0.31  total vs no problems:  ꭓ2= 1.541, df= 1, p= 0.21 |
| **Improve (%)** | 47.5 | 28 |  | 60.4 | 70 |  | 60.4 | 77.8 |  | 58.7 | 73.3 |  |
| **Worsen (%)** | 22 | 13 |  | 15.1 | 10 |  | 12.5 | 6.7 |  | 17.4 | 13.3 |  |
| **Mixed change (%)** | 84.3 | 59 |  | 71.6 | 76.9 |  | 66.7 | 75 |  | 67.6 | 77.6 |  |
| **Total with problems (%)** | 15.7 | 11 |  | 28.4 | 23.1 |  | 33.3 | 25 |  | 32.4 | 22.4 |  |
| **No problems (%)** | 30.5 | 18 |  | 24.5 | 20 |  | 27.1 | 15.6 |  | 23.9 | 13.3 |  |
| Health status | **Baseline vs. 2 weeks** | | | **Baseline vs. 4 weeks** | | | **Baseline vs. 8 weeks** | | | **Baseline vs. 12 weeks** | | |
|  | **ED-5D-5L anxiety / depression** | | | | | | | | | | | |
|  | **SSRI** | **T-XR** | **Chi2** | **SSRI** | **T-XR** | **Chi2** | **SSRI** | **T-XR** | **Chi2** | **SSRI** | **T-XR** | **Chi2** |
| **No change (%)** | 38.2 | 26 | no change, improve, worsen: ꭓ2= 0.04, df = 2, p= 0.98  total vs no problems:  ꭓ2= 0.4, df= 1, p= 0.53 | 27.4 | 23.8 | no change, improve, worsen: ꭓ2= 0.744, df= 2, p= 0.69  total vs no problems:  ꭓ2= 0.488, df= 1, p= 0.48 | 11.4 | 10.3 | no change, improve, worsen: ꭓ2= 2.94, df= 2, p= 0.23  total vs no problems:  ꭓ2= 0.034, df= 1, p= 0.85 | 21.2 | 7 | no change, improve, worsen: ꭓ2= 4.934, df= 2, p= 0.08  total vs no problems:  ꭓ2= 0.199, df= 1, p= 0.66 |
| **Improve (%)** | 52.9 | 36 |  | 67.1 | 73 |  | 77.1 | 86.2 |  | 69.7 | 82.5 |  |
| **Worsen (%)** | 8.8 | 6 |  | 5.5 | 3.2 |  | 11.4 | 3.4 |  | 9.1 | 10.5 |  |
| **Mixed change (%)** | 97.1 | 68 |  | 98.6 | 96.9 |  | 97.2 | 96.7 |  | 97.1 | 98.3 |  |
| **Total with problems (%)** | 2.9 | 2 |  | 1.4 | 3.1 |  | 2.8 | 3.3 |  | 2.9 | 1.7 |  |
| **No problems (%)** | 38.2 | 26 |  | 27.4 | 23.8 |  | 11.4 | 10.3 |  | 21.2 | 7 |  |

SSRI – group receiving selective serotonin reuptake inhibitors, T–XR – group receiving trazodone extended-release formulation,
